# Molecular data reshape our understanding of the life cycles of three digeneans (Monorchiidae and Gymnophallidae) infecting the bivalve, *Donax variabilis*: it’s just a facultative host!

**DOI:** 10.1051/parasite/2021027

**Published:** 2021-04-09

**Authors:** Kristina M. Hill-Spanik, Claudia Sams, Vincent A. Connors, Tessa Bricker, Isaure de Buron

**Affiliations:** 1 Department of Biology 205 Fort Johnson Road, College of Charleston Charleston 29412 SC USA; 2 Department of Biology, Division of Natural Sciences, University of South Carolina Upstate 1800 University Way Spartanburg 29303 SC USA

**Keywords:** Coquina, Trematode, *Lasiotocus*, *Parvatrema*, Complex life cycle evolution, DNA sequencing, Western Atlantic

## Abstract

The coquina, *Donax variabilis*, is a known intermediate host of monorchiid and gymnophallid digeneans. Limited morphological criteria for the host and the digeneans’ larval stages have caused confusion in records. Herein, identities of coquinas from the United States (US) Atlantic coast were verified molecularly. We demonstrate that the current GenBank sequences for *D. variabilis* are erroneous, with the US sequence referring to *D. fossor*. Two cercariae and three metacercariae previously described in the Gulf of Mexico and one new cercaria were identified morphologically and molecularly, with only metacercariae occurring in both hosts. On the Southeast Atlantic coast, *D. variabilis*’ role is limited to being a facultative second intermediate host, and *D. fossor,* an older species, acts as both first and second intermediate hosts. Sequencing demonstrated 100% similarities between larval stages for each of the three digeneans. Sporocysts, single tail cercariae, and metacercariae in the incurrent siphon had sequences identical to those of monorchiid *Lasiotocus trachinoti*, for which we provide the complete life cycle. Adults are not known for the other two digeneans, and sequences from their larval stages were not identical to any in GenBank. Large sporocysts, cercariae (*Cercaria choanura*)*,* and metacercariae in the coquinas’ foot were identified as *Lasiotocus choanura* (Hopkins, 1958) n. comb. Small sporocysts, furcocercous cercariae, and metacercariae in the mantle were identified as gymnophallid *Parvatrema* cf. *donacis*. We clarify records wherein authors recognized the three digenean species but confused their life stages, and probably the hosts, as *D. variabilis* is sympatric with cryptic *D. texasianus* in the Gulf of Mexico.

## Abstract (fr)

La telline, *Donax variabilis,* est un hôte intermédiaire connu de digènes Monorchiidae et Gymnophallidae. Le nombre limité de critères morphologiques pour identifier les hôtes et les stades larvaires des parasites sont à la base de confusion dans la littérature. Dans cette étude nous avons identifié par séquençage les tellines collectées sur la côte Atlantique des États-Unis. Nous démontrons que les séquences pour *D. variabilis* dans GenBank sont incorrectes et que celle du spécimen américain est en fait celle de *D. fossor*. Deux cercaires et trois métacercaires décrites au préalable dans le Golfe du Mexique ainsi qu’une nouvelle cercaire ont été identifiées morphologiquement et par séquençage. Les métacercaires seules infectent les deux hôtes. Sur la côte sud-est Atlantique, *D. variabilis* a un rôle limité à seulement celui de second hôte intermédiaire facultatif, et *D. fossor,* une espèce plus ancienne, est premier et second hôte intermédiaire. Le séquençage a montré 100 % de similarité entre les stades larvaires de chacun des trois digènes. Des sporocystes avec des cercaires à queue simple et les métacercaires dans le siphon inhalant ont des séquences identiques à celles de *Lasiotocus trachinoti*, pour qui nous donnons le cycle complet. Les adultes des deux autres digènes ne sont pas connus et les séquences des stades larvaires ne sont identiques à aucune dans GenBank. Des sporocystes de grande taille, les cercaires (*Cercaria choanura*) et les métacercaires enkystées dans le pied des tellines sont identifiées comme étant *Lasiotocus choanura* (Hopkins, 1958) n. comb. Des petits sporocystes avec des furcocercaires et des métacercaires dans le manteau des tellines sont identifiées comme étant le gymnophallide *Parvatrema* cf. *donacis*. Nous clarifions les rapports de la littérature dans lesquels les auteurs ont reconnu les trois espèces de digènes mais ont confondu leurs stades larvaires et probablement les hôtes étant donné que *D. variabilis* vit en sympatrie avec l’espèce cryptique *D. texasianus* dans le Golfe du Mexique.

## Introduction

**Figure 1:**
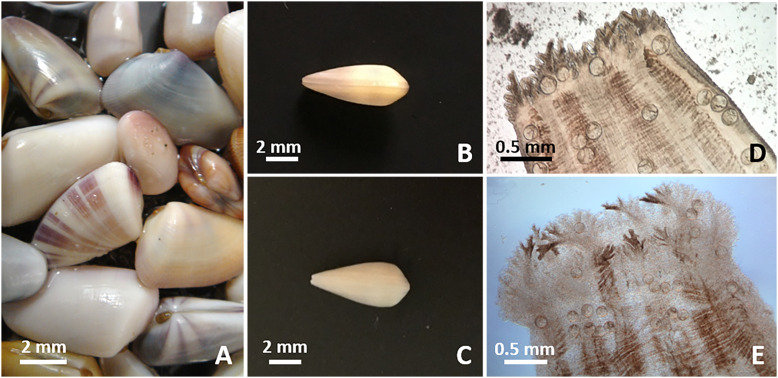
*Donax* spp. from the southeastern US Atlantic coast. (A) Coquinas showing shell color variation. (B–C) Ventral view of shell of *D. variabilis* (B) showing an elongated outline and thin anterior pedal region, and *D. fossor* (C) showing a more triangular outline compared to *D. variabilis* and a thick anterior edge. (D–E) Inhalant siphon of *D. variabilis* (D) showing smaller folds harboring smaller papillae than those of *D. fossor* (E).

Coquinas, *Donax* spp., are small hard-shell clams that live in large colonies in the surf zone of sandy beaches. Their life span is short, they are fast developing, and they are ecologically significant as they are filter feeders that are preyed upon by numerous species of crustaceans, shore birds, and marine fishes [11, 33, 36, 65]. In particular, *D. variabilis* Say, 1822 is common on the southeastern US Atlantic coast (south of Chesapeake Bay to mid-east Florida) and throughout the Gulf of Mexico from southwest Florida, USA to Campeche State, Mexico [2, 66]. It has been reported as host to a variety of parasites [in 4, 32] including several digenean larvae [29, 30, 38, 62, 63]. However, because these clams live in sympatry with congeneric cryptic species [1, 2], such host reports are questionable. Furthermore, while species names were often attributed to the cercarial stages (e.g., *Cercaria choanura* Hopkins, 1958 [30]; *Cercaria fragosa* Holliman, 1961 [29]), no association between sporocysts, cercariae, and metacercariae could be made accurately at the time of those descriptions due to limitations in morphological characters common to these stages [9]. Similarly, no connection could be established between these larval stages and adults, which infect shore birds and/or marine fishes. Hence, none of the monorchiid and gymnophallid species reported from coquinas in the Gulf of Mexico have known life cycles, and the suprageneric species are *species inquirendae* until their identity is resolved [9]. In coastal South Carolina (SC), coquinas *D. variabilis* and *D. fossor* are cryptic species, and preliminary opportunistic observations over the past decade revealed that infection by digeneans was common although unreported (Fig. 1). Using morphology, we identified two monorchiids and one gymnophallid previously described from *D. variabilis* in the Gulf of Mexico, and we discovered one previously unreported cercaria. The advent of molecular identification via DNA sequencing has made the unraveling of complex parasite life cycles much easier compared to experimental infections (e.g., [10, 40, 67]). The use of molecular tools allowed us to connect the various life cycle stages encountered, to identify cryptic intermediate hosts, and to elucidate the complete life cycle of one of the monorchiid species by identifying the adult stage from local fish, and thus, in part clarifying the current state of confusion. Further, we found that *D. variabilis* was not the sole intermediate host in the life cycles of these parasites, with, in contrast, its role limited to being a facultative, second intermediate host.

## Material and methods

### Collection of hosts and parasites

A total of 1095 coquinas were sampled opportunistically in the months of June through October between 2018 and 2020 at low tide from the swash zone along Folly Beach (32°66′34 N, 79°91′99 W) and Sullivan’s Island (32°76′60 N, 79°82′09 W) on the SC coast. Some specimens were haphazardly collected between 2010 and 2015 from the same localities, but not included in prevalence calculations. Additional coquinas were collected from North Wildwood, New Jersey (NJ) (39°00′00 N, 74°78′70 W) on September 6, 2020. Adult digeneans were collected from Florida Pompano *Trachinotus carolinus* (L.) in October 2014 at the same localities in SC and were identified and sequenced (see below) in an attempt to match resulting sequences with those from larval stages found infecting coquinas.

Coquina specimens were maintained on a bed of sand in aerated seawater in the laboratory and examined within 3 days post-collection. After the clams were shucked, gonads, digestive gland, mantle, foot, and both inhalant and exhalant siphons were isolated, squashed separately on individual slides, and examined for infection under a compound microscope. The inner side of the shells were also examined under the dissecting microscope. Clam tissue (uninfected gonad and adductor muscle) was fixed in 95% ethanol or directly frozen at −20 °C (SC), or whole clams were fixed in 100% EtOH (NJ) for molecular study. Parasites from SC coquinas and fish were isolated in filtered seawater, and parasites from NJ were isolated from the EtOH-fixed clams. There was no attempt to observe the natural emergence of cercariae out of the coquinas. Some specimens were examined and photographed fresh while others, including manually-excysted metacercariae, were heat-killed by slowly passing a flame under the slides on which they were placed. Parasites were fixed in either 95% ethanol or 5% neutral buffered formalin (NBF) for molecular and morphological studies, respectively.

### Morphological study

Fixed parasitic stages were stained with Semichon’s acetocarmine or Mayer’s hematoxylin, dehydrated according to standard protocol, and mounted in Canada balsam or Permount. Measurements were taken using an ocular micrometer and are reported in micrometers unless otherwise specified; ranges are provided with averages in parentheses. We used the morphological criteria of shell outline and size of folds and papillae of the incurrent siphon described by Simone and Dougherty [56] to verify molecular identification of the clams (see below; Fig. 1). Some clams were photographed prior to dissections and their shells were kept. Prevalence of infection was determined for *Donax* spp. because not all clam hosts were systematically identified to the species level. Once adult specimens collected from the local Florida Pompano were morphologically identified as *Lasiotocus trachinoti* Overstreet and Brown, 1970 [47]*,* we compared them to paratype USNM # 70816 to validate their identification.

Voucher specimens of parasites and clams were deposited at the Museum National d’Histoire Naturelle, Paris, France under the numbers MNHN-IM-2019-1683–2019-1687 (clam shells) and MNHN-HEL 1484–1499 (parasites) and at the National Museum of Natural History, Smithsonian Institution, USA, under the numbers USNM 1642286–1642292 (clam shells) and USNM 1642266–1642285 (parasites).

### Molecular study

#### DNA extraction

Genomic DNA of the clam hosts and parasite specimens was extracted using the DNeasy Blood and Tissue kit (Qiagen, Hilden, Germany) based on the manufacturer’s protocol, with the exception of decreasing the elution volume to 100 μL for the parasites. Parasite DNA was concentrated to ~50 μL using an Eppendorf VacufugePlus (Hamburg, Germany) prior to PCR.

#### Parasite PCR and sequencing

Portions of nuclear and mitochondrial genes were amplified and sequenced for comparison to one another and to those in the National Center for Biotechnology Information (NCBI) GenBank database. Primers and primer sequences used for each locus are in Table 1. Primers 3S [12] or GA1 [5] and ITS2.2 [20] were used to amplify the second internal transcribed spacer (ITS2) region of the ribosomal RNA (rRNA) gene of digeneans. For the 3S + ITS2.2 PCR, a 20-μL total reaction contained 0.5× GoTaq Flexi PCR Buffer (Promega, Madison, WI, USA), 2 mM MgCl_2_, 0.4 mM dNTPs, each primer at 0.4 μM, 0.06 U μL^−1^ Promega GoTaq DNA polymerase, and 1 μL template DNA. PCR cycling conditions were done as in Cutmore et al. [22]: 1 cycle of 95 °C for 3 min, 45 °C 2 min, 72 °C for 90 s, 4 cycles of 95 °C for 45 s, 50 °C for 45 s, 72 °C for 90 s; 30 cycles of 95 °C for 20 s, 52 °C for 20 s, 72 °C for 90 s; and a final extension at 72 °C for 5 min. For the GA1 + ITS2.2 PCR, a 25-μL total reaction contained 1× Promega GoTaq Flexi PCR Buffer, 1.5 mM MgCl_2_, 0.2 mM dNTPs, each primer at 0.5 μM, 0.05 U μL^−1^ Promega GoTaq DNA polymerase, and 3 μL template DNA. PCR cycling conditions were as follows: a 15-s initial denaturation at 94 °C; 32 cycles of 94 °C for 30 s, 56 °C for 30 s, 68 °C for 51 s; and a final extension at 68 °C for 3 min. Primers LSU5 and 1200R [32] or 28S_300F and 28S_ECD2 [59] were used to amplify a portion of the large subunit (28S) rRNA gene. For the LSU5 + 1200R PCR, a 25-μL total reaction contained 1× Promega GoTaq Flexi PCR Buffer, 1.5 mM MgCl_2_, 0.2 mM dNTPs, each primer at 0.4 μM, 0.05 U μL^−1^ Promega GoTaq DNA polymerase, and 1 μL template DNA. PCR cycling conditions were as follows: 5 min at 95 °C; 35 cycles of 94 °C for 30 s, 55 °C for 45 s, and 72 °C for 2 min; 72 °C for 5 min. For the 28S_300F + 28S_ECD2 PCR, a 20-μL total reaction contained 1× GoTaq Flexi PCR Buffer (Promega), 1.5 mM MgCl_2_, 0.2 mM dNTPs, each primer at 0.2 μM, 0.05 U μL^−1^ Promega GoTaq DNA polymerase, and 1 μL template DNA. PCR cycling conditions were as follows: 3 min at 94 °C; 35 cycles of 94 °C for 30 s, 55 °C for 30 s, and 72 °C for 1 min; 72 °C for 2 min. For amplification of partial cytochrome c oxidase I mitochondrial DNA (COI), a 25-μL total reaction contained 1× Promega GoTaq Flexi PCR Buffer, 3.5 mM MgCl_2_, 0.2 mM dNTPs, each primer at 0.5 μM, 0.04 U μL^−1^ Promega GoTaq DNA polymerase, and 5 μL template DNA. Cycling conditions were done as in Van Steenkiste et al. [61]: a 2-min initial denaturation at 94 °C was followed by 3 cycles at 94 °C for 40 s, 51 °C for 40 s, 72 °C for 1 min; 5 cycles 94 °C for 40 s, 50 °C (−1 °C/cycle) for 40 s, 72 °C for 1 min; 35 cycles at 94 °C for 40 s, 45 °C for 40 s, 72 °C for 1 min; and a final extension at 72 °C for 5 min.

**Table 1:** Primer and randomly amplified polymorphic DNA (RAPD) sequences used in this study.

Target	Marker	Primer name	Primer sequence (5′–3′)	Reference
Parasite	ITS2	3S	GGTACCGGTGGATCACGTGGCTAGTG	[12]
		ITS2-2	CCTGGTTAGTTTCTTTTCCTCCGC	[20]
		GA1	AGAACATCGACATCTTGAAC	[5]
		ITS2-2	CCTGGTTAGTTTCTTTTCCTCCGC	[20]
	28S	28S_300F	CAAGTACCGTGAGGGAAAGTTG	[59]
		28S_ECD2	CTTGGTCCGTGTTTCAAGACGGG	[59]
		LSU5	TAGGTCGACCCGCTGAAYTTAAGCA	[32]
		1200R	GCATAGTTCACCATCTTTCGG	[32]
	COI	Dice1F	TTWCNTTRGATCATAAG	[46]
		Dice11R	GCWGWACHAAATTTHCGATC	[61]
Host	COI	LCO1490Mba	GTAGAACTAAYCATAARGATATTGG	[43]
		HCO2198Mba	TAAACTTCTGGGTGRCCAAAAAAYCA	[43]
	RAPD	E18	GGACTGCAGA	[1]

ITS2 = second internal transcribed spacer region of the ribosomal RNA (rRNA) gene, 28S = large subunit rRNA gene, COI = cytochrome c oxidase I mitochondrial DNA.

All products were electrophoresed on 1% agarose gels (100 V, 30 min), subsequently stained with GelRed (Biotium, Inc., Hayward, CA, USA), and visualized under UV light. PCR products were cleaned using ExoSAP-IT (Affymetrix, Santa Clara, CA, USA), following the manufacturer’s protocol and sent to Eurofins MWG Operon LLC (Louisville, KY, USA) for direct bi-directional sequencing using the same primers as above. Complementary sequences were compared to one another and to their chromatograms using Sequencher version 5.4 (Gene Codes, Ann Arbor, MI, USA). Sequence alignments were performed in MEGA X [34] using MUSCLE [25]. Alignments were trimmed so that all sequences were of equal length with no terminal gaps, and *p*-distance calculations were also done in MEGA X [34] with 1000 bootstrap replicates. For ITS2 alignments, Gblocks was used to remove poorly aligned positions and divergent regions [14]. A portion of the small subunit (18S) rRNA gene was also amplified and sequenced using primers 600F and A27R [37], but sequences from the two monorchiid species were only 0.3% different from one another in a 356-bp alignment and thus not useful in differentiating the two monorchiid species found. While the 18S sequences obtained were deposited into GenBank (see Supplementary Tables 1 and 2), we did not pursue sequencing for this marker.

For the phylogenetic analysis of monorchiid 28S data, we aligned the *Lasiotocus* sequences from this study with those from other monorchiids in GenBank (namely those used in the phylogeny of Wee et al. [64] with some exceptions due to lack of overlap with sequences generated herein) and sequences from sister family Lissorchiidae (to be used as an outgroup) using MUSCLE [25]. Indels larger than 5 bases and affecting >10% of the sequences were removed, resulting in an 848-bp alignment. Maximum parsimony analysis was conducted in MEGA X [34] with 1000 bootstrap replicates and 100 random additions.

#### Host PCR, sequencing, RAPD

All coquinas infected with sporocysts (except one collected in 2018 that was lost and 5 collected in 2010–2015; see Supplementary Tables 1 and 2) and subsamples of those infected with metacercariae in the foot, siphon, and/or mantle were identified molecularly. We amplified and sequenced a portion of COI using primers LCO1490Mba and HCO2198Mba [43] for a total of 65 hosts. A 20-μL total reaction contained 1× Promega GoTaq Flexi PCR Buffer, 2.5 mM MgCl_2_, 0.2 mM dNTPs, 0.25 mg mL^−1^ bovine serum albumin (BSA), each primer at 0.5 μM, 0.05 U μL^−1^ Promega GoTaq DNA polymerase, and 3 μL template DNA. The PCR protocol was as follows: 5 min at 95 °C; 35 cycles of 95 °C for 1 min, 40 °C for 1 min, 72 °C for 1 min; and 72 °C for 7 min. Products were electrophoresed, visualized, purified, and sequenced as above. Sequences were also edited as above and compared to sequences available in the NCBI database using BLAST [3].

There were two COI sequences from *D. variabilis* in GenBank: one obtained from a specimen collected in New York (NY), USA (MH012241; [43]) and another from Mauritius (MN178795; [direct submission]); however, these sequences were only 81% similar to one another. Given this dissimilarity, we assumed that the appointed organism name of the sequence from Mauritius was an error, as this locality is outside this species’ geographic range [2]. Unexpectedly, however, the NY *D. variabilis* sequence (MH012241) was 99–100% similar to sequences of specimens we identified morphologically as *D. fossor*, but only 83–84% similar to sequences of specimens that we identified as *D. variabilis*, which themselves were 78–81% similar to other *Donax* spp. COI sequences in GenBank (KY951446, MF668317, MT334589). The discrepancy between the sequence from the specimens we identified morphologically as *D. variabilis* and the NY *D. variabilis* COI sequence in GenBank led us to suspect yet another error in the identity of the specimen associated with this GenBank sequence. This contention was further supported by the fact that the specimen used to generate the *D. variabilis* COI sequence in GenBank was collected much further north [43] than the northernmost range limit of this species [2, 56]. To confirm this suspected error, specimens of *D. fossor* were collected from NJ, USA (see above), where it occurs in the absence of other *Donax* species [2]. Ten individuals were dissected, their larval digeneans collected, and genomic DNA of clam tissues (*n* = 6) and of the parasites was extracted as described above. PCR and sequencing also were performed as above.

Additionally, we further verified the host species identity using a random amplified polymorphic DNA (RAPD) marker following the protocol of Adamkewicz and Harasewych [1]. Marker E18 (Table 1) is diagnostic for *D. variabilis*, producing a 500-bp product [1]. A 25-μL total reaction contained 1× Fermentas Buffer + KCl (Vilnius, Lithuania), 1 mg mL^−1^ BSA, 1.9 mM MgCl_2_, 0.25 mM dNTPs, primer at 0.5 μM, 0.05 U μL^−1^ Promega GoTaq DNA polymerase, and 1 μL (15 ng) template DNA. Cycling was as follows: 94 °C for 5 min followed by 45 cycles of 94 °C for 1 min, 36 °C for 1 min, 72 °C for 2 min, and a final extension at 72 °C for 5 min. Products were electrophoresed on 2% agarose gel stained with GelRed alongside a 100 bp ladder (IBI Scientific, Dubuque, IA, USA) at 75 V, 60 min and visualized under UV light. Four individuals of each *Donax* species were assayed.

## Results

### Morphological study of the parasites

#### *Lasiotocus trachinoti* Overstreet and Brown, 1970 [47] (Monorchiidae)

**Figure 2:**
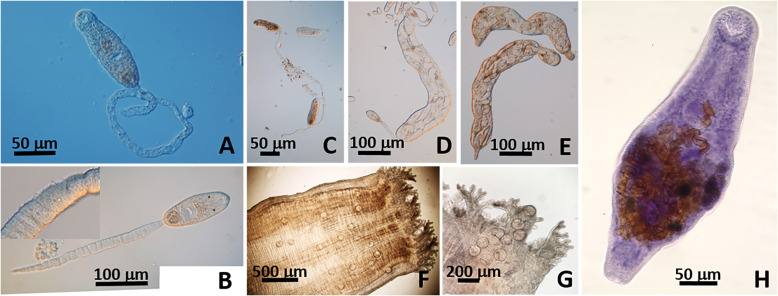
*Lasiotocus trachinoti.* (A–C) Cercaria distome with a pair of ocelli and a long retractile and spinose tail (B insert). (D–E) Large sporocysts with terminal birth pore. (F–G) Metacercariae *in situ* in inhalant siphon in *D. variabilis*. (H) Adult (Mayer’s hematoxylin) from intestine of Florida Pompano, *Trachinotus carolinus*.

Cercariae (Figs. 2A–2C): Bi-ocellate, distome, simple tail. Body 110–153 (131) long, 33–63 (50) wide at level of acetabulum (*n* = 10) not including tail. Tail spinose, retractile, often entangled and broken, 423 longest measured, 12–17 (13) wide (*n* = 5). Oral sucker 23–47 (33) in diameter (*n* = 9); mouth subterminal; pharynx 13 long, 17 wide (*n* = 1); esophagus and ceca not visible. Acetabulum in posterior half of boy, 20–43 (35) from posterior of oral sucker, 27–37 (32) long, 20–35 (30) wide (*n* = 6). One ocellus rectangular 3 × 3 on each side and slightly posterior of pharynx. Excretory bladder with thick walls 30–37 (34) long, 20–28 (24) wide, at base of tail (*n* = 5). Excretory duct extending forward, reaching anterior level of pharynx (*n* = 1). Development in elongated sporocysts in gonads. Sporocysts 500–820 (600) long, 100–167 (124) wide (*n* = 7), with terminal birth pore (Figs. 2D–2E).

Host: *Donax fossor* Say, 1822 (Donacidae)

Site of infection: Gonads

Locality: Folly Beach, South Carolina, USA

Prevalence of infection: 1.2% (13 of 1095 individual *Donax* spp. examined)

Vouchers: MNHN-HEL 1486–1489; USNM 1642273–1642277

Metacercariae (Fig. 2F–2G): Encysted, fresh heat-killed, under coverslip: 106–144 (123) long × 94–138 (115) wide (*n* = 44). Excysted in permanent preparations: Body 148–232 (181) long, 43–87 (64) wide at level of acetabulum (*n* = 9), covered with minute spines. Oral sucker 30–43 (36) in diameter (*n* = 7); mouth subterminal; pharynx 22 long, 28 wide (*n* = 1); esophagus 18 long (*n* = 1). Ceca not visible. Acetabulum at mid body, 42–73 (58) from posterior of oral sucker, 25–37 (33) long, 20–40 (30) wide (*n* = 6). Ocelli not visible on mounted specimens.

Hosts: *Donax variabilis* Say, 1822 and *D. fossor* Say, 1822 (Donacidae)

Site of infection: Inhalant siphon

Locality: Folly Beach, South Carolina, USA

Other locality: North Wildwood, New Jersey, USA

Prevalence of infection in SC: 85.5% (936 of 1095 individual *Donax* spp. examined)

Vouchers: MNHN-HEL 1484–1485, 1491; USNM 1642278, 1642279

Adult (Fig. 2H) (based on 5 individuals): Body 363–483 (423) long, 110–167 (137) wide, covered with spines. Oral sucker subterminal 40–60 (52) long, 43–53 (49) wide. Pharynx 20–30 (25) long, 28–30 (29) wide. Acetabulum at mid body, 33–73 (54) long, 37–83 (57) wide. Genital atrium 38–53 (45) long, 37–47 (43) wide with genital pore median pre-acetabulum.

Site of infection: stomach

Host: Florida Pompano *Trachinotus carolinus* (L.) (Carangidae)

Locality: Folly Beach, South Carolina, USA

Prevalence of infection: 100% (*n* = 5)

Voucher: MNHN-HEL-1490; USNM 1642280, 1642281

**Figure 3:**
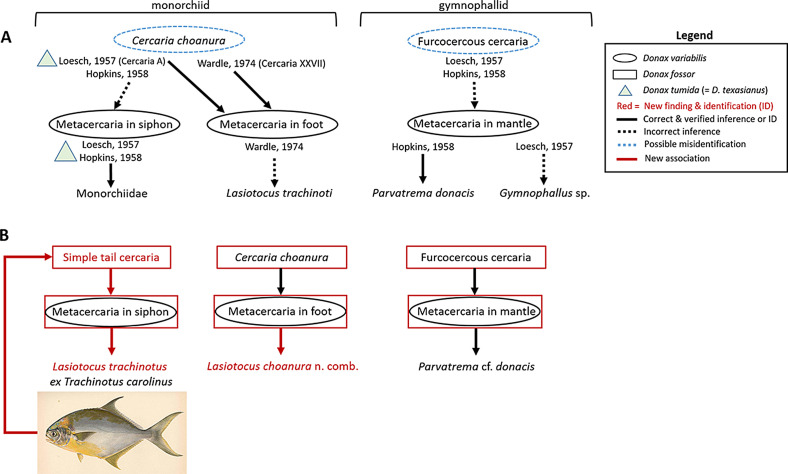
Summary of findings by authors who observed monorchiid (a) and gymnophallid (b) life stages in coquina *Donax variabilis,* barring *Cercaria pocillator* and *Cercaria fragosa*, which we did not encounter and were only speculated to encyst in *D. variabilis* by Holliman [29]. (A) previous studies; (B) Current study showing that both *Donax fossor* and *D. variabilis* may act as second intermediate host, but *D. fossor* is the sole first intermediate host on the Atlantic coast. The fish image was provided by the Freshwater and Marine Image Bank https://digitalcollections.lib.washington.edu/digital/collection/fishimages/search.

**Figure 4:**
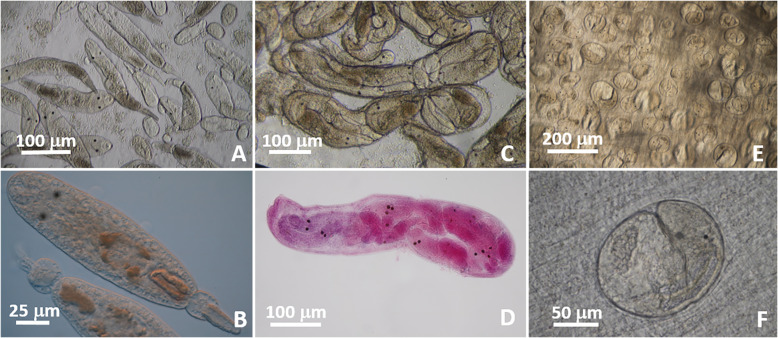
*Lasiotocus choanura* n. comb. in coquina *Donax* spp. (A–B) Live, unstained cercaria bi-ocellate, distome, with collar-like tail in *D. fossor*. (C–D) Sporocysts containing cercariae with conspicuous ocelli ((D) stained with acetocarmine). (E–F) Metacercariae, sometimes in high density (E) showing conspicuous ocelli, *in situ* in foot of coquina *D. variabilis*.

Remarks: No simple tail cercaria has been reported from coquinas in the Gulf of Mexico. In SC, sporocysts and cercariae were found only in individuals of *D. fossor*. The cercaria herein resembles that of *Postmonorchis donacis* Young, 1953 [68], which was described on the Pacific coast from the bean clam, *D. gouldii* Dall, 1921. Both cercariae are spinose and have a simple tail, a pair of ocelli, and a globular “sac-shaped” posterior excretory vesicle. However, cercariae of *L. trachinoti* have a significantly smaller body (without tail: 131 vs. 340 in *Po. donacis*), a much longer tail (423 vs. 170), and a thick walled excretory bladder. Metacercariae in the siphon of *D. variabilis* were reported by Hopkins [30], and while larger in all dimensions, resembled the ones we found. However, he erroneously suggested that they were encysted cercariae *Cercaria choanura* (a cercaria with a collar-like tail that we also report below) (Figs. 3, 4A, 4B). Wardle [62], who correctly suggested that the monorchiid *L. trachinoti* infected individuals of *D. variabilis,* also mistakenly associated *C. choanura* with this species (Fig. 3). Adults found herein fit the description of *L. trachinoti* from its type host, *T. carolinus* (see [47]).

#### *Lasiotocus choanura* (Hopkins, 1958 [30]) n. comb. (Monorchiidae)

Cercariae (Figs. 4A–4B): Bi-ocellate, distome, collar-like tail. Body 187–293 (227) long, 47–77 (58) wide at level of acetabulum (*n* = 17). Tail collar cup-like shaped 18–30 (22) long, 20–30 (25) wide (*n* = 10), with protusible portion 37–53 (47) long, 13–20 (17) wide when extended beyond collar (*n* = 9), striated. Oral sucker 28–38 (34) long, 28–37 (33) wide; mouth subterminal; prepharynx 20 long (*n* = 1); pharynx 12–18 (15) in diameter (*n* = 4); ceca very short 15 long (*n* = 1), bifurcating post pharynx. Acetabulum in posterior half of body 23–40 (30) long, 18–33 (26) wide (*n* = 14). One ocellus on each side of pharynx; rectangular, 10 long and 8 wide (*n* = 9). Excretory bladder elongate 58–82 (67) long, 27–37 (31) wide, thick walled. Genital primordium, conspicuous, between top of excretory vesicle and acetabulum. Development in tubular sporocysts in gonads. Sporocysts 627–1100 (880) long, 120–187 (147) wide; containing about 15–20 cercariae with conspicuous ocelli both in fresh and in mounted and stained specimens (Fig. 4C–4D).

Host: *Donax fossor* Say, 1822 (Donacidae)

Site of infection: Gonads

Locality: Folly Beach, South Carolina, USA

Prevalence of infection: 1.8% (20 of 1095 individual *Donax* spp. examined)

Voucher: MNHN-HEL 1495, 1498, 1499, USNM 1642266—1642269

Metacercariae (Figs. 4E–4F): encysted, fresh heat-killed, under coverslip: 113–206 (170) long × 106–194 (154) wide (*n* = 36); excysted in permanent preparations: body 257–293 (273) long, 57–87 (65) wide, covered with minute spines. Oral sucker 27–47 (39) long, 33–40 (37) wide. Pharynx 20–30 (24) long, 10–13 (11) wide (*n* = 4). Acetabulum in posterior half of body, 30–43 (37) long, 23–37 (31) wide (*n* = 3). Excretory vesicle 63 long, 33 wide (*n* = 1). Ocelli conspicuous in both fresh and mounted specimens.

Hosts: *Donax variabilis* Say, 1822 and *D. fossor* Say, 1822 (Donacidae)

Site of infection: Foot

Locality: Folly Beach, South Carolina, USA

Other locality: North Wildwood, New Jersey, USA

Prevalence of infection in SC: 75.7% (829 of 1095 individual *Donax* spp. examined)

Voucher: MNHN-HEL 1496, 1497; USNM 1642270, 1642271

Adult: unknown

Remarks: Two bi-ocellate, distome cercariae with protusible collar-like tails have been described from *D. variabilis*, both from the Gulf of Mexico: *Cercaria choanura* [30] (same as Cercaria A of Loesch [38] according to Hopkins [30]) and *C. pocillator* [29]. The adult stage of neither species is known. Herein, sporocysts and cercariae were found only in individuals of *D. fossor* (Fig. 3). Cercariae are smaller in all dimensions compared to both previously described cercariae, but most closely resemble *C. choanura* in having a conspicuous genital primordium, which *C. pocillator* lacks, and in having very short ceca. We could not observe penetration glands. Based on our observations, which are further supported by molecular data (see below), *C. choanura* is a valid taxon belonging to *Lasiotocus* Looss, 1907. We thus propose the new combination *Lasiotocus choanura* (Hopkins, 1958) n. comb. Although Holliman [29] stated that *C. pocillator* “may encyst in the first intermediate host”, he did not observe metacercariae in the clams that he examined. Hopkins [30] and Loesch [38] incorrectly attributed metacercariae they found in high prevalence in the inhalant siphon of *D. variabilis* to *C. choanura* (Fig. 3). Therefore, based on our results, these authors have confused the two species of monorchiids that we have identified. Loesch [38] did report the presence of “A” type metacercariae (associated with cotylocercous Cercaria A) in the foot of the coquinas that were examined, and Wardle [62] inferred that metacercariae in the foot of the coquina were encysted cercariae (Cercaria XXVII), which, based on the presence of short ceca, closely resembled *C. choanura*. Our results confirm these suggestions. However, Wardle [62] further suggested that this species could be the monorchiid *L. trachinoti* from the Florida Pompano, which our results showed was not the case (see above; Fig. 3). Significantly, both Loesch [38] and Hopkins [30] reported metacercariae in specimens of *D. tumida* Philippi, 1849 (now *D. texasianus* Philippi, 1847; [17, 45]).

#### *Parvatrema* cf. *donacis* Hopkins, 1958 [30] (Gymnophallidae)

**Figure 5:**
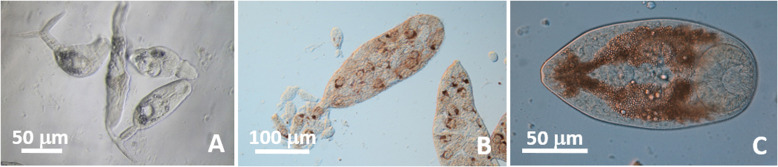
*Parvatrema* cf. *donacis* life stages in coquina *Donax* spp. (A) Unstained, live furcocercous, distome cercariae in *D. fossor*. (B) Unstained, live sporocysts with terminal birth pore in *D. fossor*. (C) Unstained, live metacercaria from mantle of *D. variabilis*.

Cercariae (Fig. 5A): Furcocercous. Body 72–120 (88) long, 27–40 (35) wide at level of acetabulum (*n* = 6). Oral sucker 25–27 (26) in diameter; mouth subterminal. Prepharynx very short; pharynx 17 long, 15 wide (*n* = 1); ceca not visible. Acetabulum 18–20 (19) long, 13–20 (17) wide, in posterior half of the body (*n* = 3). Excretory bladder U-shaped, each arm with bulge at anterior margin of acetabulum. Excretory tubules not visible. Tail stem 28–45 (34) long from point of attachment to posterior notch between furcae (*n* = 5). Furcae 22–38 (26) long, 5 (5) wide at base (*n* = 4). No spine or bristle observed on body or tail. Development in whitish sporocysts in gonads of coquina host. Sporocysts 220–353 (289) long, 80–153 (106) wide (*n* = 20). Number of embryos in all stages of development in sporocyst, 5–20; birth pore terminal (Fig. 5B).

Host: *Donax fossor* Say, 1822 (Donacidae)

Site of infection: Gonads

Locality: Folly Beach, South Carolina, USA

Prevalence of infection: 0.18% (2 of 1095 individual *Donax* spp. examined)

Voucher: MNHN-HEL 1493, 1494; USNM 1642282, 1642283

Metacercariae (Fig. 5C): Unencysted. Body 167–212 (186) long, 77–125 (101) wide at acetabulum level (*n* = 11), covered with minute spines. Oral sucker, subterminal, 48–77 (61) long, 50–73 (63) wide (*n* = 10); pharynx 20–35 (23) in diameter (*n* = 7). Acetabulum 20–38 (30) long, 22–33 (28) wide (*n* = 10). Genital pore conspicuous; distance from genital pore to anterior edge of acetabulum 17–33 (21) (*n* = 4). Distance between posterior edge of oral sucker and anterior edge of acetabulum 33–57 (44) (*n* = 11). Ceca short, blind, thick walled, 33–63 (52) long, 18–42 (31) wide (*n* = 9). Testes, symmetrical, 30–40 (33) long, 15–38 (24) wide (*n* = 7). Ovary pretesticular 15–36 (23) in diameter.

Host: *Donax variabilis* Say, 1822 and *D. fossor* Say, 1822 (Donacidae)

Site of infection: Mantle

Locality: Folly Beach, South Carolina, USA

Other locality: North Wildwood, New Jersey, USA

Prevalence of infection in SC: 0.4% (4 of 1095 individual *Donax* spp. examined)

Voucher: MNHN-HEL 1492; USNM 1642284, 1642285

Adult: unknown

Remarks: Loesch [38] called the metacercariae and furcocercous cercariae he encountered in specimens of *D. variabilis* from the Gulf of Mexico (Texas) *Gymnophallus* metacercariae and *Gymnophallus* cercariae, respectively. Hopkins [30] revisited these identifications and described the metacercariae as being *Parvatrema donacis* (Fig. 3); he also inferred that the *Gymnophallus* cercariae of Loesch [38] were cercariae of *Pa. donacis.* Holliman [29] described another gymnophallid cercaria, *Cercaria fragosa*, from individuals of *D. variabilis* in the Gulf of Mexico (Florida) and differentiated it from the cercaria described by Hopkins [30], mainly by the presence of bristles and spines on its body and tail and by the large number of embryos (over 50) per sporocyst compared to about 6 in putative sporocysts of *Pa. donacis*. Herein, we found sporocysts and cercariae only in *D. fossor*.

The presence of a conspicuous genital pore located at a distance from the anterior edge of the ventral sucker in metacercariae allowed the identification of a species of *Parvatrema* Cable, 1953 [19]. Although smaller in all dimensions, metacercariae in *D. fossor* and *D. variabilis* and cercariae in *D. fossor* from SC closely resemble those of *Pa. donacis* [30]. In particular, the number of embryos per sporocyst in our specimens was between 5 and 20 and neither spines nor bristles were observed on the body and tail of cercariae. Therefore, based on metacercaria and cercaria morphology, we identify these three stages as *Pa.* cf. *donacis* until DNA sequences of *Pa. donacis* from the type locality in the Gulf of Mexico are available.

### Molecular study

#### *Lasiotocus trachinoti* Overstreet and Brown, 1970

Sequences obtained from adult specimens (*n* = 8) from Florida Pompano were identical to those from the metacercariae found in the inhalant siphon of coquinas of both species (*n* = 11) and the simple-tailed cercariae with sporocysts (*n* = 13) based on alignments of ITS2 (312 bp), 28S (893 bp), and COI sequences (433 bp) (Tables 2 and 3; Supplementary Tables 1 and 2). Intraspecific sequence variation among COI sequences ranged from 0 to 0.1%, but appeared random and not host- or life-stage dependent; the other markers did not demonstrate any variation. ITS2 and 28S sequences were 100% similar to those of *L. trachinoti* from an adult in the Gulf of Mexico (MN984478), and COI sequences were 85.1% similar/14.9% dissimilar to that of *Lasiotocus mulli* (Stossich, 1883) (MT665981, 308-bp alignment). Sequences were deposited into GenBank and accession numbers can be found in Supplementary Tables 1 and 2.

**Table 2:** GenBank accession numbers (acc. no.) of cytochrome c oxidase I mitochondrial DNA sequences from individuals of *Donax fossor* infected by sporocysts and/or metacercariae of *Lasiotocus trachinoti*, *L. choanura*, and *Parvatrema* cf. *donacis* collected from South Carolina (SC) and New Jersey (NJ, in bold), USA from June through October between 2018 and 2020.

Acc. no.		Sporocysts			Metacercariae		
		*Lasiotocus trachinoti*	*Lasiotocus choanura*	*Parvatrema* cf. *donacis*	*Lasiotocus trachinoti*	*Lasiotocus choanura*	*Parvatrema* cf. *donacis*
MW628241		**SEQ**	**○**	**○**	**○**	**○**	**○**
MW628242		**SEQ**	**○**	**○**	**●**	**●**	**○**
MW628243		**SEQ**	**○**	**○**	**●**	**●**	**○**
MW628244		**SEQ**	**○**	**○**	**●**	**●**	**○**
MW628245		**SEQ**	**○**	**○**	**SEQ**	**●**	**○**
MW628246		**SEQ**	**○**	**○**	**●**	**●**	**○**
MW628247		**SEQ**	**○**	**○**	**●**	**●**	**○**
MW628248		**SEQ**	**○**	**○**	**●**	**●**	**○**
MW628249		**SEQ**	**○**	**○**	**●**	**●**	**○**
*MW628250*		**SEQ**	**○**	**○**	**●**	**●**	**○**
MW628251		**SEQ**	**○**	**○**	**●**	**●**	**○**
MW628252		**●**	**○**	**○**	**●**	**●**	**○**
MW628253		**●**	**●**	**○**	**●**	**●**	**○**
*MW628254*		**○**	**SEQ**	**○**	**SEQ**	**SEQ**	**○**
MW628255		**○**	**SEQ**	**○**	**●**	**SEQ**	**○**
MW628256		**○**	**SEQ**	**○**	**●**	**SEQ**	**○**
MW628257		**○**	**SEQ**	**○**	**●**	**●**	**○**
MW628258		**○**	**SEQ**	**○**	**○**	**●**	**○**
MW628259		**○**	**●**	**○**	**●**	**●**	**○**
MW628260		**○**	**●**	**○**	**●**	**●**	**○**
MW628261		**○**	**●**	**○**	**●**	**●**	**○**
MW628262		**○**	**●**	**○**	**●**	**●**	**○**
MW628263		**○**	**●**	**○**	**●**	**●**	**○**
MW628264		**○**	**●**	**○**	**●**	**●**	**○**
MW628265		**○**	**●**	**○**	**●**	**●**	**○**
MW628266		**○**	**●**	**○**	**●**	**●**	**○**
MW628267		**○**	**●**	**○**	**●**	**●**	**○**
MW628268		**○**	**●**	**○**	**●**	**●**	**○**
MW628269		**○**	**●**	**○**	**●**	**●**	**○**
MW628270		**○**	**●**	**○**	**●**	**●**	**○**
MW628271		**○**	**●**	**○**	**●**	**●**	**○**
MW628272		**○**	**●**	**○**	**●**	**●**	**○**
MW628273		**○**	**○**	**SEQ**	**●**	**●**	**○**
MW628274		**○**	**○**	**○**	**●**	**●**	**○**
MW628275		**○**	**○**	**○**	**●**	**●**	**○**
MW628276		**○**	**○**	**○**	**●**	**●**	**○**
MW628277		**○**	**○**	**○**	**●**	**●**	**○**
MW628278		**○**	**○**	**○**	**●**	**●**	**SEQ**
*MW628279*		**○**	**○**	**○**	**SEQ**	**●**	**○**
MW628280		**○**	**○**	**○**	**●**	**●**	**○**
MW628281		**○**	**○**	**○**	**○**	**●**	**SEQ**
MW628282		**○**	**○**	**○**	**●**	**●**	**○**
**MW628283**		**○**	**○**	**○**	**●**	**SEQ**	**SEQ**
**MW628284**		**○**	**○**	**○**	**●**	**SEQ**	**SEQ**
**MW628285**		**○**	**○**	**○**	**●**	**●**	**SEQ**
**MW628286**		**○**	**○**	**○**	**●**	**SEQ**	**○**
**NO SEQ**		**○**	**○**	**○**	**SEQ**	**●**	**○**
**MW628287**		**○**	**○**	**○**	**○**	**SEQ**	**○**
Total	48	13	20	1	44	47	5
No. seq. (SC)	42	11	5	1	3	3	2
No. seq. (NJ)	5	0	0	0	1	4	3
Total seq.	47	11	5	1	4	7	5

● = presence of parasite, ○ = absence of parasite, SEQ = parasite was sequenced (see Supplementary Table 1), italicized GenBank accession numbers indicate that sequencing was performed in only one direction.

**Table 3:** GenBank accession numbers (acc. no.) of cytochrome c oxidase I mitochondrial DNA sequences from individuals of *Donax variabilis* infected by metacercariae of *Lasiotocus trachinoti*, *L. choanura*, and *Parvatrema* cf. *donacis* collected from South Carolina, USA from June through October between 2018 and 2020 (except for two individuals (*) that were collected in 2010 and not included in prevalence data). No individual was infected with sporocysts.

Acc. no.		Sporocysts (all species)	Metacercariae		
			*Lasiotocus trachinoti*	*Lasiotocus choanura*	*Parvatrema* cf. *donacis*
MW628288		○	●	●	●
MW628289		○	**SEQ**	**SEQ**	○
*MW628290*		○	●	●	○
*MW628291*		○	●	●	○
*MW628292*		○	●	●	○
MW628293		○	**SEQ**	**SEQ**	○
MW628294		○	●	**SEQ**	○
MW628295		○	●	●	○
MW628296		○	**SEQ**	**SEQ**	○
MW628297		○	**SEQ**	**SEQ**	○
*MW628298*		○	●	●	○
MW628299		○	●	●	○
MW628300		○	●	●	○
MW628301		○	●	●	○
MW628302		○	○	●	○
MW628303		○	○	●	○
MW628304*		○	○	●	●
MW628305*		○	○	●	●
Total	18	0	14	18	3
Total seq.	18	0	4	5	0

● = presence of parasite, ○ = absence of parasite, SEQ = parasite was sequenced (see Supplementary Table 1), italicized GenBank accession numbers indicate that sequencing was performed in only one direction.

#### *Lasiotocus choanura* (Hopkins, 1958) n. comb

**Figure 6:**
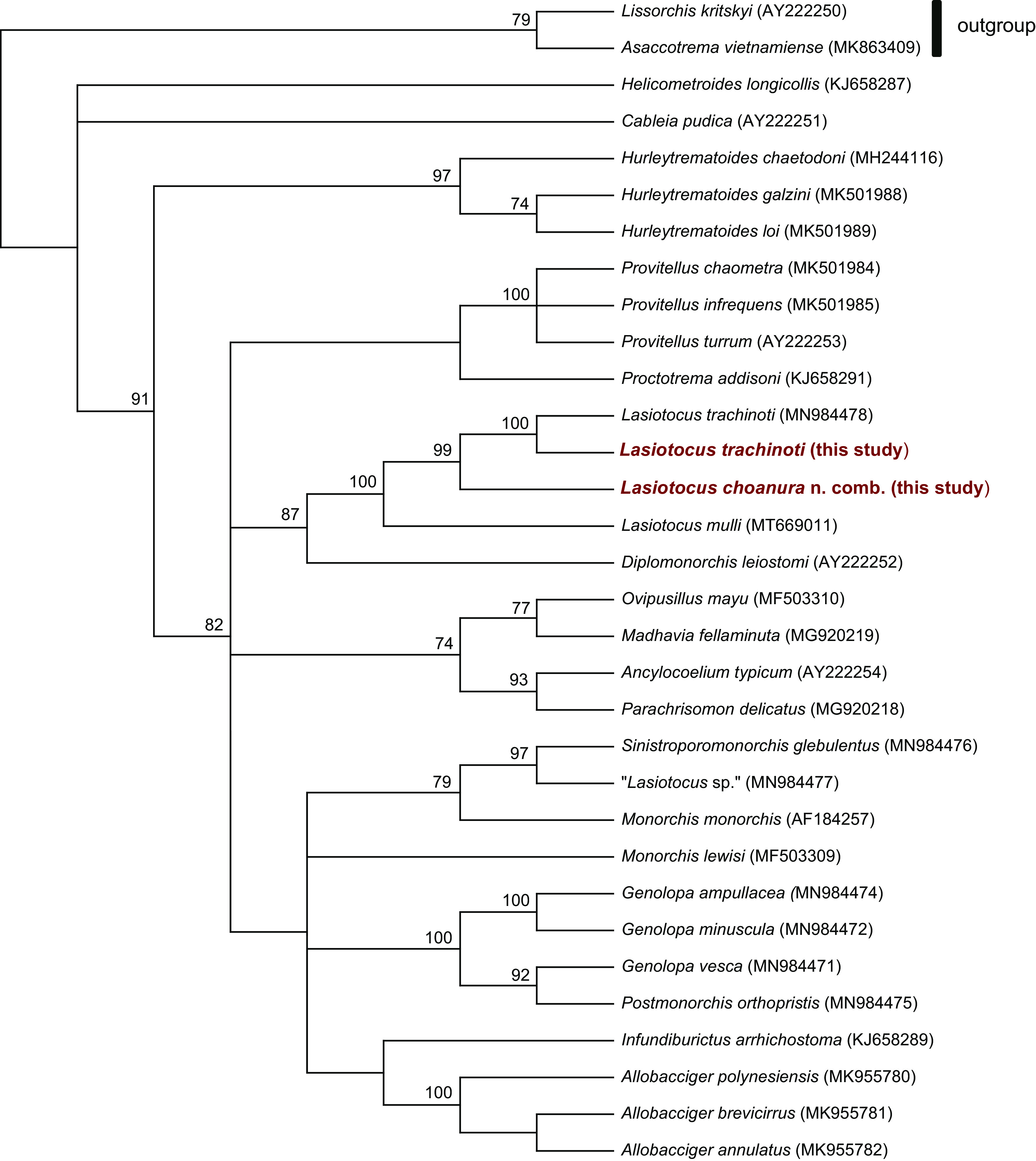
Phylogenetic relationships among monorchiid taxa based on maximum parsimony analysis of an 848-base pair alignment of partial large subunit (28S) ribosomal RNA gene sequences from the current study (in red and bolded) and from GenBank. Bootstrap support values are shown above the nodes (values < 70% are not shown and branches with < 50% support are collapsed).

Sequences from metacercariae found in the foot of coquinas of both species (*n* = 19) were identical to those from *C. choanura* with sporocysts (*n* = 7) based on alignments of ITS2 (304 bp), 28S (870 bp), and COI sequences (433 bp) (Tables 2 and 3; Supplementary Tables 1 and 2). Intraspecific sequence variation among COI sequences ranged from 0 to 0.5%, but again appeared random and not host- or life-stage dependent; the other markers did not show any intraspecific variation. The ITS2 sequence from this species differed from that of *L. trachinoti* ITS2 sequence by 1.3% based on a 304-bp alignment, 1.7% based on a 870-bp alignment of 28S sequences, and 8.1% based on a 433-bp alignment of COI sequences. ITS2, 28S, and COI sequences differed from *L. mulli* sequences by 13.4% (352 bp), 6.6% (870 bp), and 13.6% (308 bp), respectively. The maximum parsimony analysis of monorchiid 28S data produced a consensus tree with a topology similar to that generated by Wee et al. [64] (Fig. 6). The placement of this species within *Lasiotocus* was based on this phylogeny where its sequence formed a well-supported monophyletic clade with those of *L. mulli* and *L. trachinoti*. Sequences were deposited into GenBank and accession numbers can be found in Supplementary Tables 1 and 2.

#### *Parvatrema* cf. *donacis* Hopkins, 1958

Sequences from metacercariae found in the mantle of coquinas (*n* = 7) were identical to those from sporocysts with furcate cercaria (*n* = 2) based on ITS2 (363 bp) and 28S (938 bp) alignments (Tables 2 and 3; Supplementary Tables 1 and 2). PCR and sequencing of a portion of the COI gene was not successful. ITS2 sequences were 93–94% similar to other *Parvatrema* spp. ITS2 sequences in GenBank and formed a monophyletic clade sister to those of *Parvatrema* spp. in GenBank (*Pa. duboisi* (Dollfus, 1923) AB478508-81, MH882785, MW193330; *Parvatrema* sp. SP-2011, JN381026; *Parvatrema* sp. CG-2014, KM246856). The partial 28S rRNA gene sequence was 91% similar to that from *Parvatrema* sp. CG-2014 (KM246856) in GenBank; no other *Parvatrema* 28S rRNA gene sequence from GenBank overlapped with sequences from this study. Sequences were deposited into GenBank and accession numbers can be found in Supplementary Tables 1 and 2.

### Host identification

**Figure 7:**
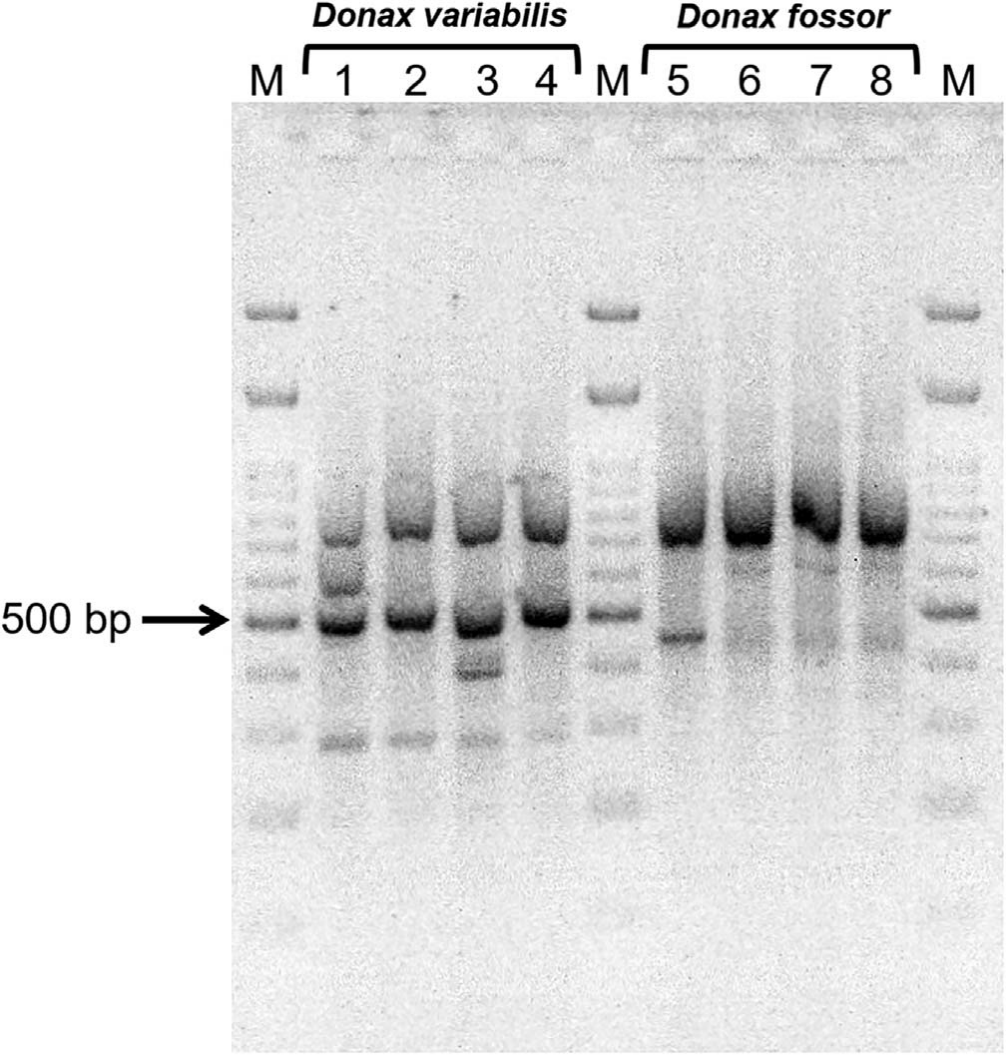
RAPD amplifications generated using primer E18 on a 2% agarose gel alongside a 100-base pair (bp) ladder (M). Lanes 1–4 are amplifications from individuals of *Donax variabilis*, and lanes 5–8 are from individuals of *D. fossor*. Only *D. variabilis* individuals produced the 500-bp diagnostic band.

As mentioned above, partial COI gene sequences of specimens morphologically identified as *D. fossor* (both from SC and NJ) were ≥99% similar to a *D. variabilis* COI sequence in GenBank (MH012241) collected from NY [43], whereas *D. variabilis* specimens had sequences that were only 83–84% similar to this NY *D. variabilis* sequence. Specimens morphologically identified as *D. variabilis* produced the diagnostic 500-bp product using RAPD marker E18, while specimens identified as *D. fossor* did not (Fig. 7), confirming the identity error of *D. variabilis* in GenBank. Coquinas infected with sporocysts of both monorchiids (*L. trachinoti n* = 13; *L. choanura* n. comb. *n* = 20) and of the gymnophallid (*n* = 1) were all identified molecularly as *D. fossor* (Table 2); note: one coquina infected by gymnophallid sporocysts and cercariae was lost and thus identified as *Donax* sp. (Supplementary Table 2). One individual *D. fossor* (MW628253, Table 2) had a double infection with sporocysts and cercariae of both monorchiid species. Identities of another 32 coquinas with metacercariae of either species were also verified molecularly, showing that both *D. variabilis* and *D. fossor* were infected by the three types of metacercariae (Tables 2 and 3; Fig. 3). Partial COI gene sequences from hosts were deposited into GenBank as accession numbers MW628241–MW628287 (*D. fossor*; Table 2) and MW628288–MW628305 (*D. variabilis*; Table 3) and voucher specimens of both species were deposited as MNHN-IM 2019-16083–2019-16085 (*D. fossor*) and MNHN-IM 2019-16086, 2019-16087 (*D. variabilis*); USNM 1642289–1642291 (*D. fossor*) and USNM 1642286–1642288 (*D. variabilis*).

## Discussion

Coquinas *D. fossor and D. variabilis* from the US southeastern Atlantic coast were found to be infected by larval stages of two monorchiid and one gymnophallid digenean species that were previously described from *D. variabilis* in the Gulf of Mexico [30, 38, 62]. One of these monorchiids was also reported to occur in *D. tumida* [30, 38], now *D. texasianus* (see [17, 45]) in the Gulf of Mexico. Significantly, however, *D. variabilis* was identified as both first and second intermediate hosts of these digeneans in the Gulf of Mexico, but neither sporocyst nor cercaria was found in this species on the SC coast, a finding brought to light only after we molecularly identified all the individual clams serving as first intermediate hosts of these three digeneans in our collections. Thus, given the long term and recurrent issue with *Donax* species misidentification [1, 2, 45], and because *D. variabilis* lives in sympatry with the cryptic species *D. texasianus* in the Gulf of Mexico [1, 2, 60], it is then highly probable that the first intermediate host from these earlier studies was in fact misidentified. This is supported by a detail in Loesch [38], who reported infection by Cercaria A (i.e., presumed *Cercaria choanura* of Hopkins [30] and *Lasiotocus choanura* herein) in *D. variabilis* in the text, but illustrated it as being from *D. tumida* (= *D. texasianus*; [17, 45]) as he found one infected individual of this species. Loesch [38] further mentioned that all individuals of *D. tumida* he examined from his collection site in Louisiana, USA and most of those from Mustang Island, Texas, USA were infected by metacercariae type A, thus indicating that infection was indeed observed in both *Donax* species. One other area of confusion from the literature is that, whereas Loesch [38], Hopkins [30], and Wardle [62] all suggested certain connections between the life stages that they observed, such inferences could only be speculative given that no experimental infection was carried out and that sporocysts and cercariae cannot be identified to species level on the sole basis of morphology. As a result, the associations of these digeneans’ larval stages proposed by these authors were also mistaken. Sequencing data allowed us to right these previous records, which we clarify herein. In a nutshell, the monorchiid *Cercaria choanura* is identified as *Lasiotocus choanura* n. comb., its metacercariae encyst in the foot of the clam, and its adults are not known. Cercariae of *L. trachinoti*, on the other hand, have a simple long retractile tail, had not been observed prior to our study, its metacercariae encyst in the inhalant siphon, and its adults infect the digestive tract of the Florida Pompano, *T. carolinus*. Cercariae of the gymnophallid *Parvatrema* cf. *donacis* are furcocercous, its metacercariae do not encyst (as is typical of gymnophallids) and infect the mantle of the coquinas, and its adult remains to be identified. Lastly, for the US Atlantic coast, *D. fossor* serves as sole first intermediate host and also as second intermediate host, while *D. variabilis* is only a second intermediate host.

Monorchiidae is a very speciose family [39, 66]; however, of the 20 species with a bivalve as intermediate host, only nine have known life cycles: one from the Mediterranean coast [41], one from the European Atlantic coast ([10]; in [40]), three from the North American Atlantic coast [42, 57, 58], two from the South American Atlantic coast [8, 28], and two from the North American Pacific coast [23, 68]. Most of these known life cycles are two species-host cycles, as is the case in *D. fossor* herein for both *Lasiotocus* species found, which include the use of the same bivalve species as both first and second intermediate host. For rare species of this family, metacercariae may be found within sporocysts [8, 10] or may be extruded into the water [57, 58]. More typically, however, metacercariae encyst in the tissues of the bivalve, similar to those we observed in *D. variabilis* and *D. fossor*. Cercariae known as *Cercaria choanura* were identified herein as *Lasiotocus choanura* n. comb. and encyst in the foot of the coquina and not in the inhalant siphon as Hopkins [30] reported. Adults have yet to be found and/or sequenced, but according to the 28S rRNA gene phylogeny, this species appears to be sister to *L. trachinoti*. Wardle [62] inferred correctly that these cercariae encyst in the foot, but incorrectly suggested that they were of *L. trachinoti,* which as adults, commonly infect the digestive tract of the Florida Pompano ([47, 48]; present study). While cercariae of *L. trachinoti* develop in coquinas, albeit *D. fossor* on the US Atlantic coast, they had not been observed prior to our study, and we thus demonstrate this cycle in its entirety. There is no significant morphological or molecular difference (based on 1TS2 and 28S sequences) between adult specimens of *L. trachinoti* from the Atlantic coast and the Gulf of Mexico, which further supports that our data can be applied to the infections of coquinas in the Gulf of Mexico. The cercariae of *L. trachinoti* have a simple long and retractile tail (similar to those of group 1 in Cremonte et al. [18]), whereas that of *L. minutus* (Manter, 1931) is microcercous [57, 58]. Hence, the cercaria type, which is typically thought to be a reliable indicator of phylogenetic relationships [21], would appear to vary within *Lasiotocus*, a discrepancy further supporting the contention that some species assignments to the genus *Lasiotocus* are problematic and need revisiting [39, 48, 55]. Wee et al. [64] began restructuring this genus, and although cercariae morphology was not taken into account in their study, it may be significant to note that *L. elongatus* (Manter, 1931), which was reassigned to the genus *Proctotrema* Odhner, 1911, has microcercous cercariae. Wee et al. [64], however, could only gather molecular data for the type species, *L. mulli,* and while these authors suggest that *Lasiotocus* needs further refinement, for lack of current knowledge in some morphological structures and molecular data, *L. trachinoti* and *L. minutus* currently remain in the same genus until further data are available. Examination of two nuclear markers showed that sequences from the two monorchiids we encountered were less than 2% dissimilar from one another, and our phylogenetic analysis of 28S rRNA gene data showed that they form a well-supported monophyletic clade with *L. mulli*, supporting their inclusion in the genus. However, the COI sequences were ~8% different between sequences from the two species and over 14% different from the *L. mulli* sequence. While there is, to our knowledge, no *a priori* species delineation for monorchiids using COI sequence data (or any other locus), the difference in COI sequences, which show little to no intraspecific variation, along with the strikingly different cercariae, lead us to question whether these monorchiid species belong in the same genus. Finding and/or molecularly identifying the adults of *Lasiotocus choanura* n. comb. as well as the cercariae of *L. mulli* would be of particular interest to clarify this conundrum.

The high prevalence of *L. trachinoti* metacercariae in the inhalant siphon of the coquinas compared to the rather rare occurrence of sporocysts in *D. fossor* supports the contention of Bagnato [8] that a long tail in cercariae suggests a free-living lifestyle. This monorchiid may have a two-host life cycle utilizing the same species of bivalve (in this case, *D. fossor*) as both first and second intermediate hosts but not the same individuals given the rarity of sporocyst-infected coquinas compared to those harboring metacercariae. Maillard [41] experimentally demonstrated such a cycle for the monorchiid, *Paratimonia gobii* Prévot & Bartoli, 1967 by infecting individuals of the bivalve *Abra ovata* (now *A. segmentum* [Récluz, 1843] – see [66]). In this case, cercariae leave the first intermediate host (an individual of *A. ovata*), only to be sucked in by another individual of *A. ovata* (the second intermediate host) within which they encyst and accumulate in the inhalant siphon. The infected siphons then become autotomic and are eaten by the fish definitive host (a goby). While we do not know if siphons infected by metacercariae of *L. trachinoti* become autotomic, observations of coquinas with incurrent siphons missing folds indicate that Florida Pompano (known predators of coquinas [6]) may also become infected by partially eating siphons without killing the clam. Because the coquinas would stay alive and accrue metacercariae, Pompano feeding in this manner would enhance these parasites’ fitness, especially combined with the occurrence of an additional second intermediate host as metacercariae also commonly infect the siphon of individuals of *D. variabilis*. Taken together, these factors would then explain the high prevalence of *L. trachinoti* on the SC coast.

Gymnophallid adult digeneans infect marine charadriiform (shorebirds) or anseriform (ducks) [19, 24, 54] and typically have a three-host life cycle with bivalves as first intermediate hosts and bivalves or, more rarely, gastropods [13] or polychaetes [35, 51] as second intermediate hosts [54]. There are also known examples of unusual cycles that involve parthenogenetic metacercariae [26, 27] or no free-living stage [31]. Less rare are cases of shortened life cycles involving the same first and second intermediate hosts (e.g., [40, 67]) as we report herein for *Pa.* cf. *donacis* in *D. fossor*. Loesch [38] reported that all 11 of the clams (presumed to be *D. variabilis*) he examined for gymnophallid metacercariae were infected, and Hopkins [30] described the gymnophallid *Pa. donacis* from metacercariae from individuals presumed to be *D. variabilis* on Mustang Island, Texas, wherein 85% of his coquinas were infected compared to the 0.4% we report herein. Hopkins [30] did not find furcocercous cercariae in any of the 100 coquinas he examined, Loesch [38] found them in 0.7% (8/1017), and we found them in only 0.18% (2/1095). Truncated life cycles have a marked seasonality when definitive hosts are migratory birds [52], which is often the case for gymnophallids [16, 67]. While all these authors focused most of their parasite observations during summer as we did, the difference in latitude (and thus the onset of seasons) between SC and the Gulf of Mexico could explain in part such discrepancies in prevalence of infection. Differences in the abundance and migratory patterns of the definitive host(s) at different localities could also be highly relevant. Although Loesch [38] did not report finding adult gymnophallids in the plovers, sanderlings, and eastern willets that he observed feed on coquinas, it appears that he only examined them for gut contents. Thus, any of these birds, as well as for instance, Ruddy turnstones that are also known migratory predators of coquinas [53], could be definitive hosts.

Complexity in parasite life cycles evolves according to cost-benefit trade-offs for the parasite [7, 15, 44]. In digeneans, which encounter repetitive constraints in the transmission of their various larval stages, life cycles often become shorter [50]. The occurrence of three species of digeneans with two host-life cycles using the same individuals of *D. fossor* as both first and second intermediate hosts may support this contention. On the other hand, the short life span and relatively limited biotope of coquinas add other constraints to parasite transmission that may be circumvented by the addition of another intermediate host, albeit facultative as in this case. Such lateral incorporation of a facultative host can provide extra fitness to the parasites, and is not unusual in digeneans [49]. Our data best support this second scenario because phylogenetic analyses indicate that *D. fossor* and *D. texasianus* (the cryptic species that occurs in the Gulf of Mexico) are both older than *D. variabilis* and display ancestral characters (smaller size, non-migratory behavior, and subtidal habitat) [2]. Since from an evolutionary standpoint, two-host cycles are thought to precede three-host cycles [21], it thus may be inferred that the two monorchiids reported herein have two-host cycles that expand to integrate a third host, either individuals of *D. fossor,* as our prevalence data indicate, or of *D. variabilis* when present. This may also occur for the gymnophallid, but we have less evidence given the low prevalence of this parasite. The non-essential role of *D. variabilis* in the life cycles of these digeneans is evident, as these cycles occur only via *D. fossor* in NJ where it is the sole *Donax* species present. The increase in complexity of these parasites’ life cycles via the addition of *D. variabilis*, when present, as a second intermediate host would be expected to alter the transmission dynamics of the parasites and affect their fitness, thereby modifying the selective pressure impacting the evolution of their life history traits.

In conclusion, the combined use of morphological and molecular tools has allowed us to correct the identity and the association of digenean life cycle stages previously described from the clam “*D. variabilis*” in the Gulf of Mexico. These findings also allowed us to narrow the role of *D. variabilis* to that of a facultative second intermediate host on the southeastern US Atlantic coast, whereas the more ancient *D. fossor* is both a first and second intermediate host in the life cycles of these three digenean species encountered. In light of these findings, and because the misidentification of *Donax* species on the US Atlantic and Gulf of Mexico coastlines has been a long-term recurrent issue, we suspect that sporocyst-infected coquinas in earlier reports were probably misidentified as *D. variabilis* and that *D. texasianus*, also older than *D. variabilis*, acts as first and second intermediate host for these parasites in the Gulf of Mexico. How the addition of *D. variabilis* in the life cycles of these digeneans impacts the transmission dynamics and whether it results in an increased fitness of these parasites will be the object of future studies.

## Supplementary materials

Supplementary material is available at https://www.parasite-journal.org/10.1051/parasite/2021027/olm *Supplementary Table 1*. GenBank accession numbers of parasite cytochrome c oxidase I (COI) mitochondrial DNA, second internal transcribed spacer region of the ribosomal RNA gene (ITS2), and partial large (28S) and small (18S) subunit ribosomal RNA gene sequences from sporocysts and metacercariae collected from *Donax* spp. and adults from carangid *Trachinotus carolinus* in South Carolina and New Jersey (in bold), USA. Italicized accession numbers are sequences that were successfully sequenced in only one direction. *Supplementary Table 2*. GenBank accession numbers for parasite cytochrome c oxidase I (COI) mitochondrial DNA, second internal transcribed spacer region of the ribosomal RNA gene (ITS2), and partial large (28S) and small (18S) subunit ribosomal RNA gene sequences from sporocysts and metacercariae from *Donax* specimens whose identity could not be verified molecularly because they were collected between 2010 and 2015 prior to discovering that *D. fossor* can be found sporadically in the intertidal zone in South Carolina with *D. variabilis*. Coquinas infected by sporocysts and cercariae of the *Lasiotocus* species was inferred to be *D. fossor* based upon extensive molecular studies since that time (Tables 2 and 3 in text). Data for *Parvatrema* cf. *donacis* sporocyst-infected *Donax* were too limited to allow us to make the same inference. Specimens listed here were not accounted for in prevalence data. Italicized accession numbers are sequences that were successfully sequenced in only one direction.
