# Supplementary material for: Molecular data reshape our understanding of the life cycles of three digeneans (Monorchiidae and Gymnophallidae) infecting the bivalve, Donax variabilis: it’s just a facultative host!
Source: Parasite. 2021 Apr 9;28:34. doi: 10.1051/parasite/2021027 (PMC8034251; doi:10.1051/parasite/2021027)
Supplement: Supplementary file 1 — Supplementary Table 1. GenBank accession numbers of parasite cytochrome c oxidase I (COI) mitochondrial DNA, second internal transcribed spacer region of the ribosomal RNA gene (ITS2), and partial large (28S) and small (18S) subunit ribosomal RNA gene sequences from sporocysts and metacercariae collected from Donax spp. and adults from carangid Trachinotus carolinus in South Carolina and New Jersey (in bold), USA. Italicized accession numbers are sequences that were successfully sequenced in only one direction. [file parasite-28-34-s1.pdf]

Supplementary Table 1. GenBank accession numbers of parasite cytochrome c oxidase I (COI) mitochondrial DNA, second internal transcribed spacer region of the ribosomal RNA gene (ITS2), and partial large (28S) and small (18S) subunit ribosomal RNA gene sequences from sporocysts and metacercariae collected from *Donax* spp. and adults from carangid *Trachinotus carolinus* in South Carolina and New Jersey (in bold), USA. Italicized accession numbers are sequences that were successfully sequenced in only one direction.

			sporocysts			metacercariae			adults			
**Parasite**	**Host**	**Host COI**	**COI**	**ITS2**	**28S**	**COI**	**ITS2**	**28S**	**COI**	**ITS2**	**28S**	**18S**
Lasiotocus trachinoti	*Donax fossor*	*MW628241*	*MW628329*	MW627657	*MW627691*	-	-	-	-	-	-	-
	Donax fossor	MW628242	*MW628330*	MW627658	MW627692	-	-	-	-	-	-	-
	Donax fossor	MW628243	*MW628326*	MW627659	-	-	-	-	-	-	-	-
	Donax fossor	MW628244	*MW628331*	MW627660	-	-	-	-	-	-	-	-
	Donax fossor	MW628245	MW628234	MW627661	MW627693	*MW628336*	-	MW627698	-	-	-	-
	Donax fossor	MW628246	*MW628332*	MW627662	-	-	-	-	-	-	-	-
	Donax fossor	MW628247	*MW628333*	MW627663	MW627694	-	-	-	-	-	-	-
	Donax fossor	MW628248	*MW628334*	MW627664	MW627695	-	-	-	-	-	-	-
	Donax fossor	MW628249	*MW628335*	MW627665	-	-	-	-	-	-	-	-
	Donax fossor	MW628250	*MW628327*	MW627666	-	-	-	-	-	-	-	-
	Donax fossor	MW628251	*MW628328*	-	-	-	-	-	-	-	-	-
	Donax fossor	*MW628254*	-	-	-	*MW628337*	MW627667	MW627696	-	-	-	-
	Donax fossor	*MW628279*	-	-	-	*MW628338*	MW627668	MW627697	-	-	-	-
	Donax fossor	-	-	-	-	-	**MW627669**	-	-	-	-	-
	Donax variabilis	MW628289	-	-	-	-	MW627670	-	-	-	-	-
	Donax variabilis	MW628293	-	-	-	-	MW627671	MW627699	-	-	-	-
	Donax variabilis	MW628296	-	-	-	-	MW627672	MW627700	-	-	-	-
	Donax variabilis	MW628297	-	-	-	-	MW627673	MW627701	-	-	-	-
	Trachinotus carolinus	-	-	-	-	-	-	-	*MN389437*	MN380323	MK804761	MN380237
	Trachinotus carolinus	-	-	-	-	-	-	-	MN389438	MN380322	MN380243	MN380236
	Trachinotus carolinus	-	-	-	-	-	-	-	-	MN380321	MN380244	MN380235
	Trachinotus carolinus	-	-	-	-	-	-	-	MN389439	MN380319	MN380245	MN380233
	Trachinotus carolinus	-	-	-	-	-	-	-	MN389440	MN380317	MN380246	MN380232
	Trachinotus carolinus	-	-	-	-	-	-	-	-	MN380312	MN380247	-
	Trachinotus carolinus	-	-	-	-	-	-	-	-	MN380311	MN380248	-
	Trachinotus carolinus	-	-	-	-	-	-	-	-	MN380310	MN380249	-
Lasiotocus choanura n.comb.	*Donax fossor*	*MW628241*	MW628235	MW627674	MW627702	MW628237	-	-	-	-	-	-
	Donax fossor	MW628255	*MW628339*	MW627675	MW627703	*MW628342*	-	-	-	-	-	-
	Donax fossor	MW628256	*MW628340*	MW627676	MW627704	MW628238	-	-	-	-	-	-
	Donax fossor	MW628257	*MW628341*	MW627677	MW627705	-	-	-	-	-	-	-
	Donax fossor	MW628258	MW628236	MW627678	*MW627706*	-	-	-	-	-	-	-
	Donax fossor	**MW628283**	-	-	-	-	**MW627679**	-	-	-	-	-
	Donax fossor	**MW628284**	-	-	-	***MW628343***	**MW627680**	**MW627707**	-	-	-	-
	Donax fossor	**MW628286**	-	-	-	-	**MW627681**		-	-	-	-
	Donax fossor	**MW628287**	-	-	-	-	**MW627682**	-	-	-	-	-
	Donax variabilis	MW628289	-	-	-	*MW628344*	-	-	-	-	-	-
	Donax variabilis	MW628293	-	-	-	*MW628345*	-	-	-	-	-	-
	Donax variabilis	MW628294	-	-	-	MW628239	MW627683	MW627708	-	-	-	-
	Donax variabilis	MW628296	-	-	-	MW628240	MW627684	MW627709	-	-	-	-
	Donax variabilis	MW628297	-	-	-	*MW628346*	-	-	-	-	-	-
Parvatrema cf. *donacis*	*Donax fossor*	MW628273	-	MW627685	MW627710	-	-	-	-	-	-	-
	Donax fossor	MW628278	-	-	-	-	MW627686	MW627711	-	-	-	-
	Donax fossor	MW628281	-	-	-	-	MW627687	MW627712	-	-	-	-
	Donax fossor	**MW628283**	-	-	-	-	**MW627688**	**MW627713**	-	-	-	-
	Donax fossor	**MW628284**	-	-	-	-	**MW627689**	**MW627714**	-	-	-	-
	Donax fossor	**MW628285**	-	-	-	-	**MW627690**	**MW627715**	-	-	-	-
