# Supplementary material for: Molecular data reshape our understanding of the life cycles of three digeneans (Monorchiidae and Gymnophallidae) infecting the bivalve, Donax variabilis: it’s just a facultative host!
Source: Parasite. 2021 Apr 9;28:34. doi: 10.1051/parasite/2021027 (PMC8034251; doi:10.1051/parasite/2021027)
Supplement: Supplementary file 2 — Supplementary Table 2. GenBank accession numbers for parasite cytochrome c oxidase I (COI) mitochondrial DNA, second internal transcribed spacer region of the ribosomal RNA gene (ITS2), and partial large (28S) and small (18S) subunit ribosomal RNA gene sequences from sporocysts and metacercariae from Donax specimens whose identity could not be verified molecularly because they were collected between 2010 and 2015 prior to discovering that D. fossor can be found sporadically in the intertidal zone in South Carolina with D. variabilis. Coquinas infected by sporocysts and cercariae of the Lasiotocus species was inferred to be D. fossor based upon extensive molecular studies since that time (Tables 2 and 3 in text). Data for Parvatrema cf. donacis sporocyst-infected Donax were too limited to allow us to make the same inference. Specimens listed here were not accounted for in prevalence data. Italicized accession numbers are sequences that were successfully sequenced in only one direction. [file parasite-28-34-s2.pdf]

Supplementary Table 2. GenBank accession numbers for parasite cytochrome c oxidase I (COI) mitochondrial DNA, second internal transcribed spacer region of the ribosomal RNA gene (ITS2), and partial large (28S) and small (18S) subunit ribosomal RNA gene sequences from sporocysts and metacercariae from *Donax* specimens whose identity could not be verified molecularly because they were collected between 2010 and 2015 prior to discovering that *D. fossor* can be found sporadically in the intertidal zone in South Carolina with *D. variabilis*. Coquinas infected by sporocysts and cercariae of the *Lasiotocus* species was inferred to be *D. fossor* based upon extensive molecular studies since that time (Tables 2 and 3 in text). Data for *Parvatrema* cf. *donacis* sporocyst-infected *Donax* were too limited to allow us to make the same inference. Specimens listed here were not accounted for in prevalence data. Italicized accession numbers are sequences that were successfully sequenced in only one direction.

**Parasite**	**Life Stage**	**COI**	**ITS2**	**28S**	**18S**	**Host**
Lasiotocus trachinoti	sporocysts and cercariae	-	MN380320	MN380250	MN380234	*Donax* cf. *fossor*
		MN389443	MN380318	MN380251	MN380238	*Donax* cf. *fossor*
	metacercariae	-	MN380313	MN380254	-	*Donax* sp.
		MN389444	MN380314	-	-	*Donax* sp.
		MN389442	MN380315	MN380253	MN380230	*Donax* sp.
		MN389441	MN380316	MN380252	MN380231	*Donax* sp.
Lasiotocus choanura n. comb	sporocysts and cercariae	MN389451	MN384227	-	MN381808	*Donax* cf. *fossor*
		MN389452	MN384228	-	MN381809	*Donax* cf. *fossor*
	metacercariae	MN389445	MN384229	MK844590	MN381806	*Donax* sp.
		MN389446	MN384230	MN381340	MN381807	*Donax* sp.
		MN389447	MN384231	MN381345	-	*Donax* sp.
		MN389448	MN384232	MN381344	-	*Donax* sp.
		MN389449	MN384233	MN381343	-	*Donax* sp.
		MN389450	MN384234	MN381342	-	*Donax* sp.
		-	MN384235	MN381341	-	*Donax* sp.
Parvatrema cf. *donacis*	sporocysts and cercariae	-	MN384713	MN384710	MN384716	*Donax* sp.
	metacercariae	-	MN384714	MN384708	MN384711	*Donax* sp.
		-	MN384715	MN384709	MN384712	*Donax* sp.
